# A Rare Case of Situs Inversus Partialis With Levocardia in a 73-Year-Old Patient

**DOI:** 10.7759/cureus.74061

**Published:** 2024-11-20

**Authors:** Omar Salaheldin Ibrahim Mahrous, Kah Cheong Tong, Osahon Andrew Gbinigie

**Affiliations:** 1 Acute Internal Medicine, Stepping Hill Hospital, Stockport, GBR

**Keywords:** congenital birth defect, dysphagia, esophageal carcinoma, situs inversus with levocardia, weight loss

## Abstract

Situs inversus partialis (SIP) is an extremely rare congenital disorder in which most of the visceral organs are located on the opposite side of their usual anatomical locations. The condition is usually associated with levocardia, in which the apex of the heart is directed toward the left side. In our case study, a female patient with a history of dysphagia and weight loss presented to the outpatient clinic under the urgent two-week wait pathway. She underwent a CT scan of the thorax, abdomen, and pelvis, which revealed that she has SIP in which some of the abdominal organs are reversed and others are in their normal anatomical locations. The patient was diagnosed with esophageal carcinoma. However, because of her rare condition, it was challenging to insert a nasogastric tube into her reversed stomach and to explore other feeding options, e.g., jejunostomy. This report discusses a rare case of SIP, incidentally discovered in a patient with suspected esophageal malignancy, and its associated surgical challenges in her management.

## Introduction

Situs inversus is a congenital condition characterized by reversed sites of the major visceral organs in the chest, abdomen, or pelvis. The condition may present in the form of situs inversus totalis (SIT), in which all of the body organs are positioned in a mirror image of the normal human anatomy, and situs inversus partialis (SIP), in which some organs are reversed and others are located in their normal anatomical positions [1]. The prevalence of SIT is very rare, occurring in approximately 1 in 20,000 to 50,000 people. SIP is even rarer with a prevalence of approximately 1 in 2,000,000 affected [2]. In SIT, the apex of the heart is usually directed to the right side, known as dextrocardia. However, in SIP, the heart remains at its normal anatomical position, known as levocardia [3]. Our case study focused on a woman presenting with dysphagia and weight loss who was incidentally discovered to have SIP.

## Case presentation

A 73-year-old female patient presented to the gastroenterology outpatient clinic with a history of dysphagia to solid food and liquids. The symptoms had started gradually several months beforehand with a progressive course and were associated with episodes of vomiting 30 minutes after eating. She also reported a weight loss of one stone over two months. There were no complaints of diarrhea, constipation, or abdominal pain. The patient had a past history of hypertension and hemorrhagic stroke two months prior to presentation with no residual neurological deficits. She had no other medical history and was a non-smoker. Physical examination revealed that the patient was vitally stable, alert, and oriented to time, place, and person. Her vitals on admission were blood pressure of 109/81, heart rate of 83, and respiratory rate of 17 with saturations of 100% on room air. No respiratory or cardiac symptoms were associated with her condition. The apex of the heart was located on the left side in the fifth intercostal space, mid-clavicular line.

Several blood tests were ordered for the patient, including a full blood count, liver function tests, and urea and electrolytes to assess her renal function. A computerized tomography scan of the thorax, abdomen, and pelvis (CT-TAP) was also requested. All of the patient's blood test results were within normal limits apart from her potassium; she had mild hypokalemia at 3.3 mmol/l and hypoalbuminemia with a serum albumin of 36 g/dl in Table 1.

**Table 1:** Laboratory investigations

Investigations		Patient's results	Normal references
Full blood count	Hemoglobin level	124 g/l	115-165 g/l
	White cell count	5 x 10^9^/L	3.7-11 x 10^9^/L
	Platelets	304 x 10^9^/L	150-450 x 10^9^/L
Liver function test	Total bilirubin	15 µmol/l	0-21 µmol/l
	Serum alanine transferase	15 U/L	0-33 U/L
	Alkaline phosphatase	64 U/l	20-130 U/L
	Serum albumin	36 g/l	38-51 g/l
Urea and electrolytes	Serum sodium	139 mmol/L	133-146 mmol/L
	Serum potassium	3.3 mmol/L	3.5-5.5 mmol/L
	Creatinine	44 µmol/L	44-97 µmol/L
	Estimated glomerular filtration rate	>90 ml/minute/1.73m	>90 ml/minute
C-reactive protein		<4.0 mg/l	0-10 mg/l

**Figure 1:**
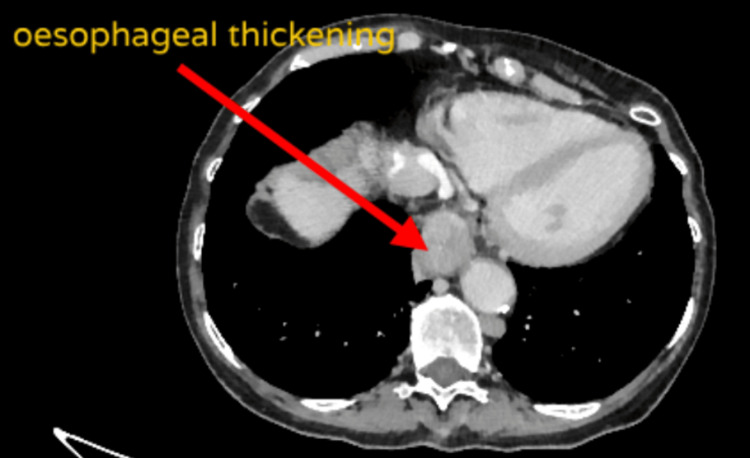
CT-TAP showing concentric esophageal thickening CT-TAP: computerized tomography scan of the thorax, abdomen, and pelvis

**Figure 2:**
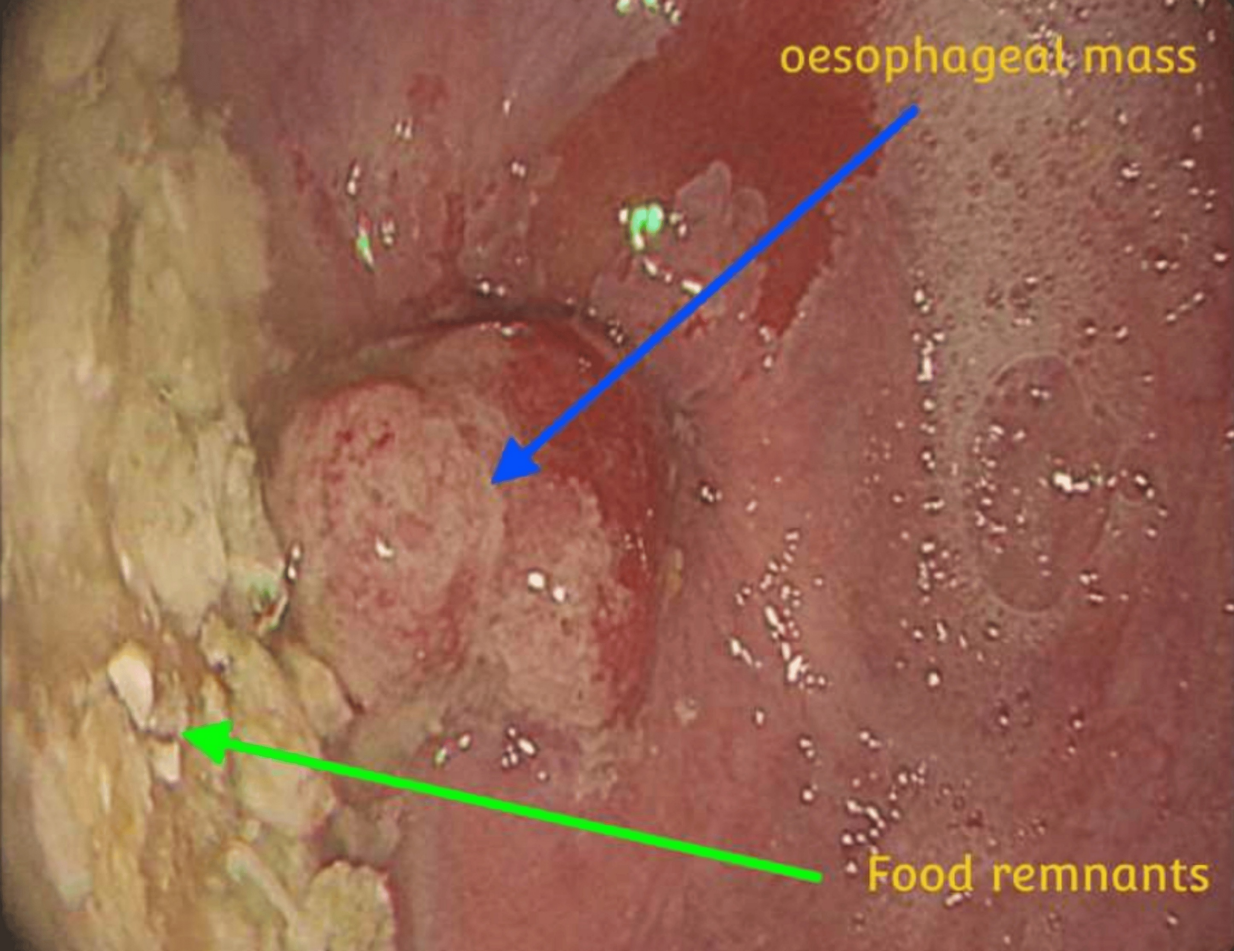
Esophagogastroduodenoscopy showing esophageal mass at the lower part of the esophagus

The CT-TAP revealed significant thickening at the lower third of the esophagus, suggesting an esophageal malignancy shown in Figure 1. Esophagogastroduodenoscopy confirmed these findings in Figure 2 and a pathology report from the biopsied lesion. The tumor was 3 cm in length, and it was impassable by the endoscope. The final diagnosis was distal esophageal carcinoma without local or distant metastasis, staging T3N0M0.

**Figure 3:**
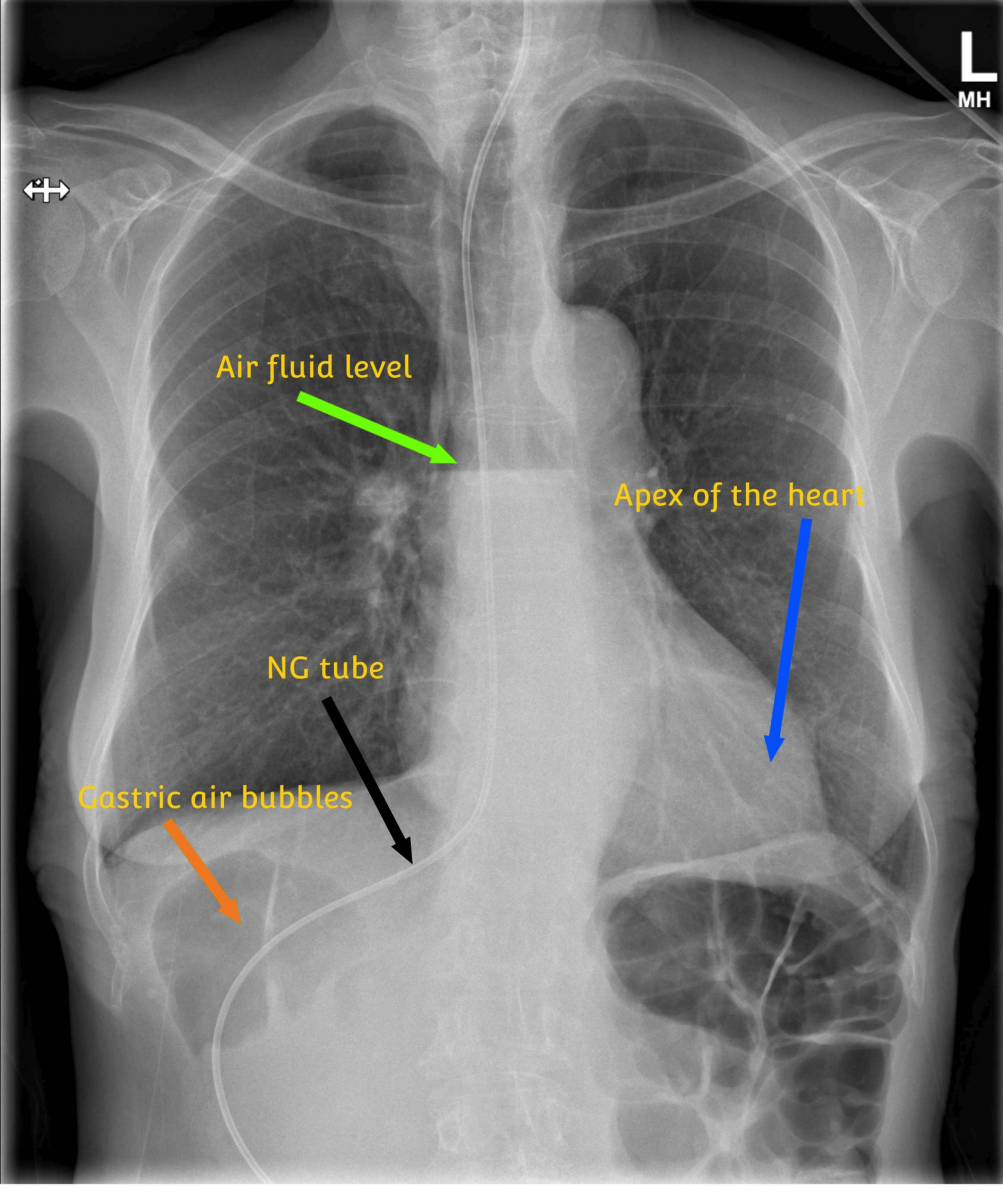
Posterior-anterior view chest X-ray showing the apex of the heart on the left side, nasogastric tube curving to the right below the diaphragm, gastric air bubbles on the right side, and an air-fluid level due to esophageal obstruction NG: nasogastric

**Figure 4:**
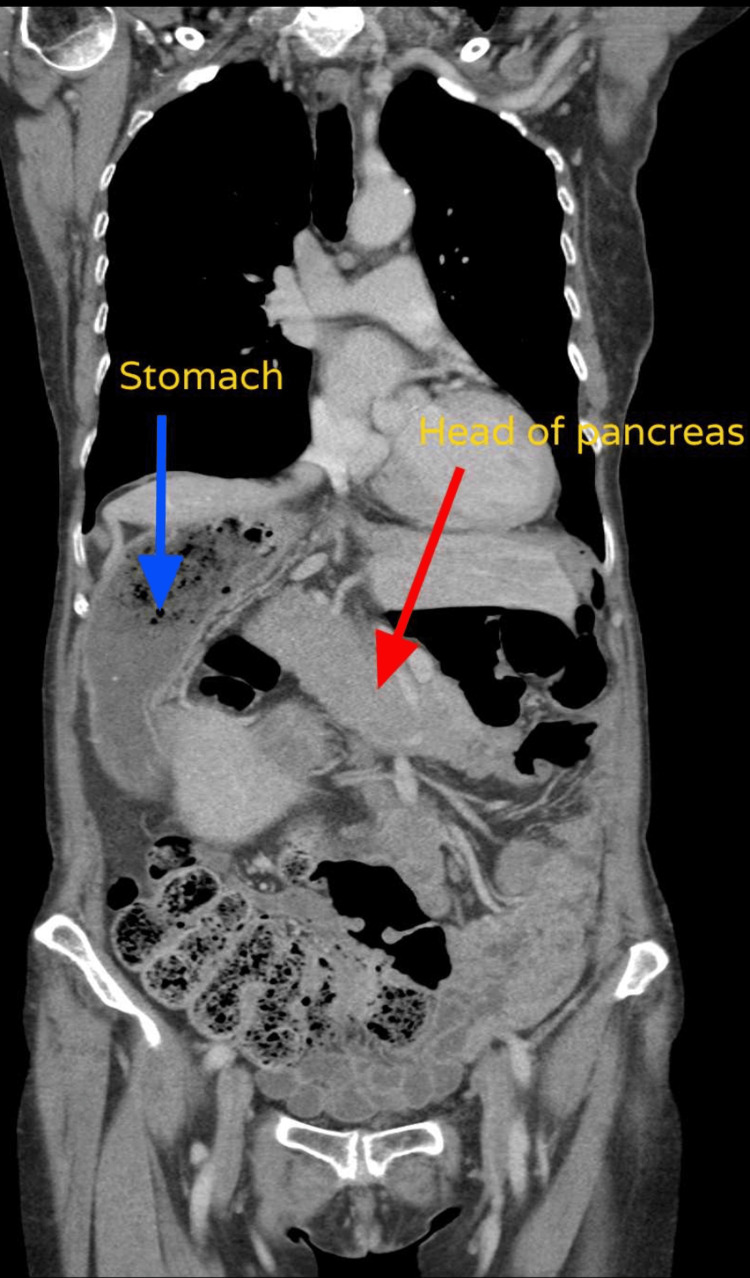
CT-TAP showing the head of the pancreas and the stomach on the right side CT-TAP: computerized tomography scan of the thorax, abdomen, and pelvis

**Figure 5:**
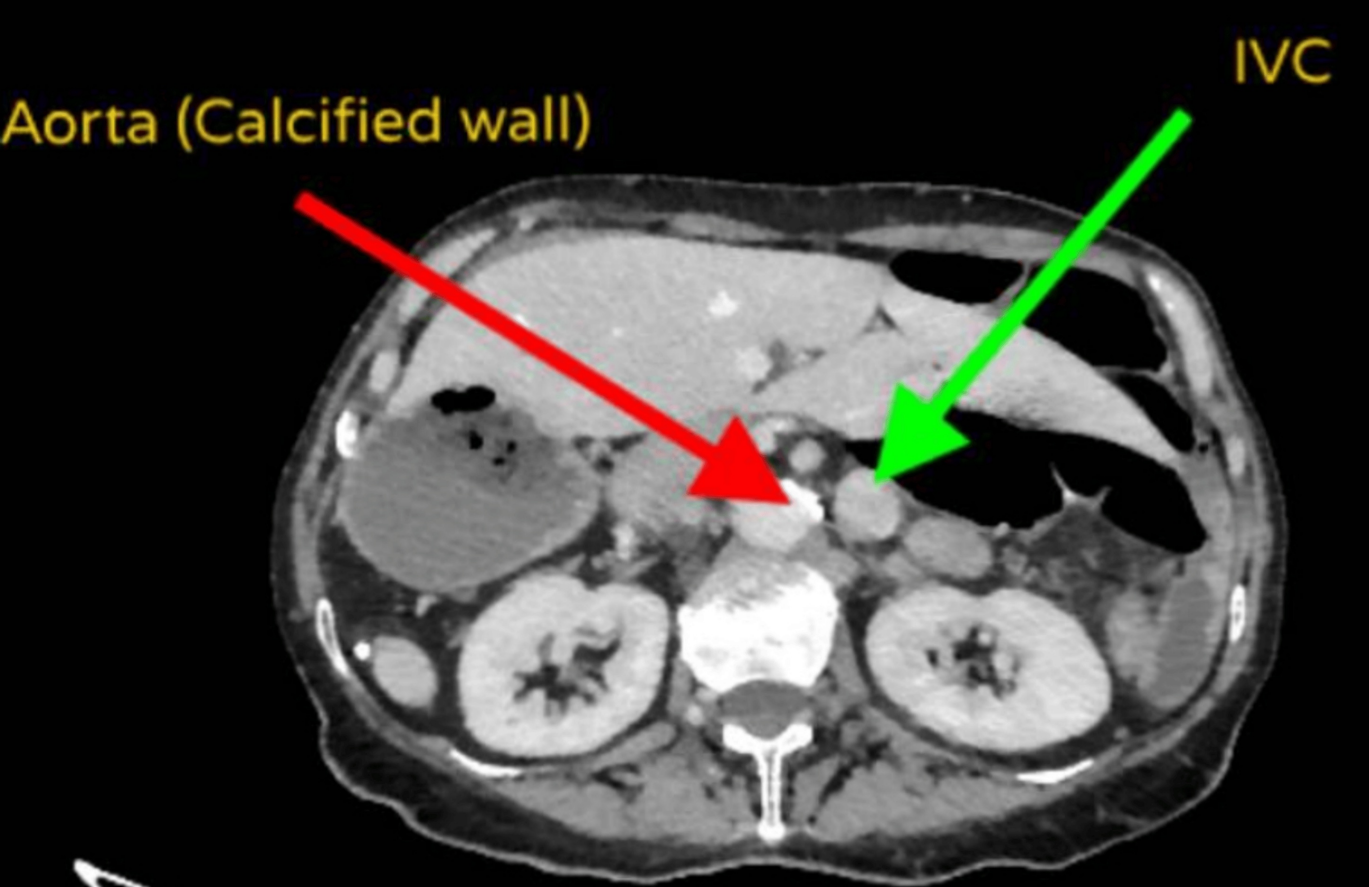
CT-TAP showing the aorta, with calcification of the aortic wall. The aorta is located on the right side and the IVC is located on the left CT-TAP: computerized tomography scan of the thorax, abdomen, and pelvis; IVC: inferior vena cava

**Figure 6:**
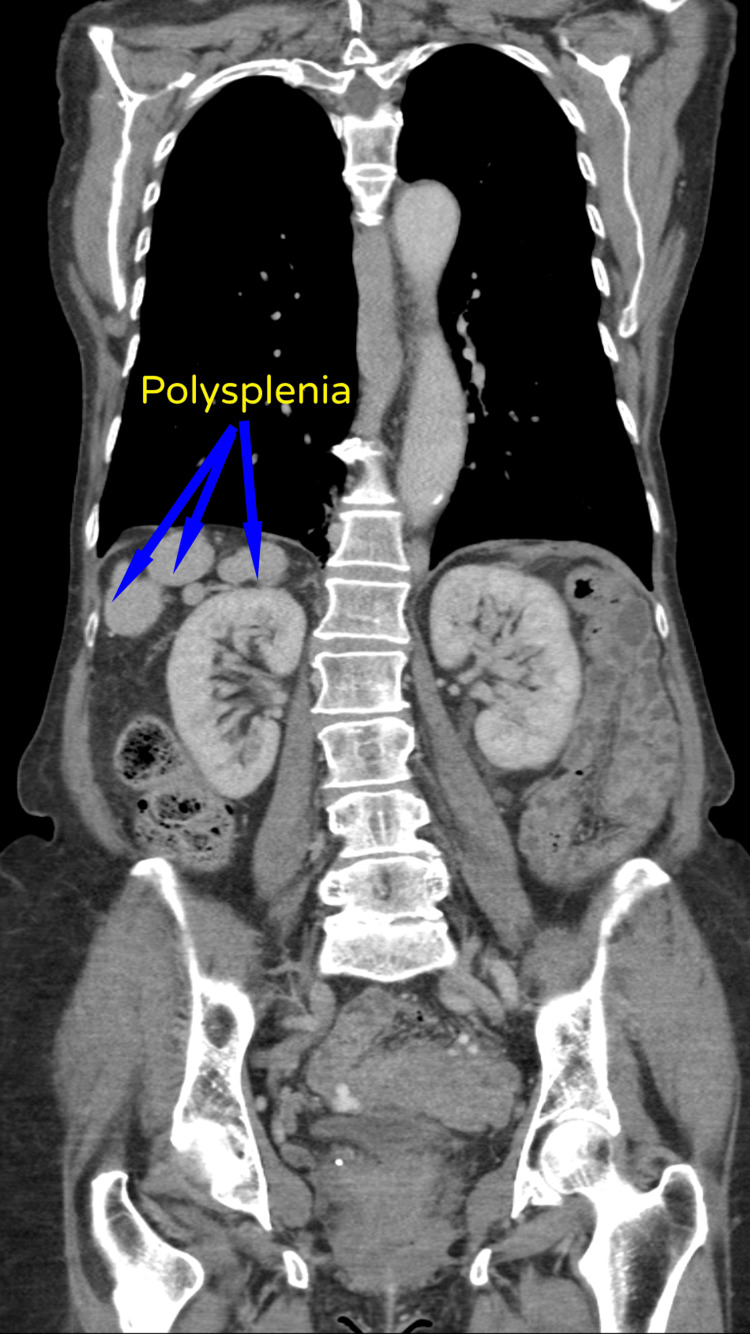
CT-TAP showing polysplenia on the right-hand side CT-TAP: computerized tomography scan of the thorax, abdomen, and pelvis

Additionally, the CT-TAP incidentally revealed SIP with the apex of the heart pointing to the left side of the body, as seen in Figure 3. The stomach was located on the right side of the body, whereas the duodenum and head of the pancreas were on the left side, as in Figure 4. The kidneys and ovaries were located in their usual anatomical positions. The inferior vena cava (IVC) was located on the left side of the abdominal aorta shown in Figure 5. Furthermore, polysplenia was present on the right side of the body, as seen in Figure 6.

Due to the patient’s unusual anatomy, inserting a nasogastric tube proved challenging. One was initially inserted by a consultant interventional radiologist, as seen in Figure 3, but was accidentally pulled out by the patient a few days later. This lady went on to have several nasogastric and nasojejunal tubes inserted. Each time the tubes became displaced and had to be reinserted by interventional radiologists. During this time the patient’s care was also complicated by an episode of pneumonia.

At the time of writing, the patient is awaiting the insertion of a radiologically inserted gastrostomy. She will no longer be having surgery and is awaiting chemoradiotherapy.

## Discussion

SIP with levocardia is a very rare congenital anomaly characterized by the arrangement of the visceral organs in a mirror image of their usual anatomical locations [3,4]. Individuals with this condition are usually asymptomatic, and their unique anatomy is often discovered incidentally during the investigations of another, unrelated medical condition [5]. In our case study, the patient presented with a history of progressive dysphagia, weight loss, and frequent episodes of vomiting. Upon performing a CT-TAP, the patient was found to have SIP. In addition, the patient’s heart was located in its normal anatomical position with no associated congenital anomalies. This is consistent with most studies that report SIP with levocardia [3,4]. However, Deolikar et al. reported a case of SIP with levocardia that had associated congenital heart disease in the form of a congenital single atrium [6]. They also reported a congenital defect in the form of a wandering spleen, and this was similar to our findings of a multilobulated spleen located underneath the right lobe of the liver. Furthermore, in our case, the stomach was located on the right side of the patient, and this was consistent with another case documented by Kanani and Sheikh, who reported a right-sided stomach during the autopsy of a female patient with suicidal poisoning [3]. Abnormalities in the IVC were also reported in the previous literature. Our patient’s IVC was located to the left of the abdominal aorta with no abnormalities in its branches, anatomical course, or divisions. This is contrary to Loukas et al., who reported a double IVC in a case of SIP [7]. Feeding is one of the most challenging problems in a patient with esophageal cancer. It was even more challenging for our SIP patient. This lady had to have several nasogastric and nasojejunal tube insertions and is awaiting the placement of a radiologically inserted percutaneous gastrostomy (RIG). Studies showed that the RIG tube is a favorable option for enteral feeding in situs inversus patients with dysphagia and normally functioning stomachs [8,9].

## Conclusions

SIP with levocardia is a very rare condition. Because of the abnormal anatomical positions of the organs, SIP's clinical significance lies in the difficulty of the diagnosis and the management of the patient should surgical intervention be required. Rare cases such as this enhance our understanding of human anatomy and shed light on the challenges in the management of any surgical conditions in similar patients with SIP. This interesting case report should also raise awareness of procedural difficulties in patients with SIP.
